# Do English NHS Microbiology laboratories offer adequate services for the diagnosis of UTI in children? Healthcare Quality Improvement Partnership (HQIP) Audit of Standard Operational Procedures

**DOI:** 10.1099/jmm.0.000114

**Published:** 2015-09

**Authors:** Cliodna A. M. McNulty, Neville Q. Verlander, Philippa C. L. Moore, James Larcombe, Jan Dudley, Jaydip Banerjee, Lyda Jadresic

**Affiliations:** ^1^​ Public Health England Primary Care Unit, Microbiology Department, Gloucestershire Royal Hospital, Great Western Road, Gloucester GL1 3NN, UK; ^2^​ Public Health England, Centre for Infectious Disease Surveillance and Control, Colindale, London, UK; ^3^​ Gloucestershire Hospitals NHS Foundation Trust, Microbiology Department, Gloucestershire Royal Hospital, Great Western Road, Gloucester GL1 3NN, UK; ^4^​ Centre for Integrated Health Care Research, University of Durham Queens Campus, Stockton-on-Tees TS17 6BH, UK; ^5^​ Bristol Royal Hospital for Children, Bristol BS2 8BJ, UK; ^6^​ University Hospitals of Leicester NHS Trust, Leicester LE1 5WW, UK; ^7^​ Gloucestershire Royal Hospital, Great Western Road, Gloucester GL1 3NN, UK

## Abstract

The National Institute of Care Excellence (NICE) 2007 guidance CG54, on urinary tract infection (UTI) in children, states that clinicians should use urgent microscopy and culture as the preferred method for diagnosing UTI in the hospital setting for severe illness in children under 3 years old and from the GP setting in children under 3 years old with intermediate risk of severe illness. NICE also recommends that all ‘infants and children with atypical UTI (including non-*Escherichia coli* infections) should have renal imaging after a first infection’. We surveyed all microbiology laboratories in England with Clinical Pathology Accreditation to determine standard operating procedures (SOPs) for urgent microscopy, culture and reporting of children's urine and to ascertain whether the SOPs facilitate compliance with NICE guidance. We undertook a computer search in six microbiology laboratories in south-west England to determine urine submissions and urine reports in children under 3 years. Seventy-three per cent of laboratories (110/150) participated. *Enterobacteriaceae* that were not *E. coli* were reported only as coliforms (rather than non-*E. coli* coliforms) by 61 % (67/110) of laboratories. Eighty-eight per cent of laboratories (97/110) provided urgent microscopy for hospital and 54 % for general practice (GP) paediatric urines; 61 % of laboratories (confidence interval 52–70 %) cultured 1 μl volume of urine, which equates to one colony if the bacterial load is 10^6^ c.f.u. l^− 1^. Only 22 % (24/110) of laboratories reported non-*E. coli* coliforms and provided urgent microscopy for both hospital and GP childhood urines; only three laboratories also cultured a 5 μl volume of urine. Only one of six laboratories in our submission audit had a significant increase in urine submissions and urines reported from children less than 3 years old between the predicted pre-2007 level in the absence of guidance and the 2008 level following publication of the NICE guidance. Less than a quarter of laboratories were providing a service that would allow clinicians to fully comply with the first line recommendations in the 2007 NICE UTI in children guidance. Laboratory urine submission report figures suggest that the guidance has not led to an increase in diagnosis of UTI in children under 3 years old.

## Introduction

Urine testing remains important to obtain a definitive diagnosis in the management of urinary tract infection (UTI), and culture and susceptibility are needed to guide antibiotic treatment. This is just as important in UTI in children as in adults. The National Institute of Health and Care Excellence (NICE) 2007 guidance CG54, on ‘Urinary tract infection (UTI) in children: diagnosis, treatment and long-term management’, states that clinicians should refer all infants under 3 months old with suspected UTI to paediatric specialist care, and a urine sample should be sent from all children with suspected UTI for urgent microscopy and culture (Box 1) (NICE, 2007). In children between 3 months and 3 years with suspected UTI, the guidance recommends that clinicians should use urgent microscopy and culture as the preferred diagnostic method over dipsticks with leukocyte esterase and nitrite. The guidance states that the dipstick can be used in this age group if urgent microscopy facilities are not available. It recommends that children under 3 months as well as children with severe illness and those with intermediate level of illness who are assessed as needing hospital care should be referred to hospital where urgent microscopy should be used (Box 1) (NICE, 2007).

**Table:** Box 1 NICE 2007 guidance (Tables 4.16–4.19) for urine testing in infants and children

**Urine testing strategy for infants younger than 3 months**		
All infants younger than 3 months with suspected UTI should be referred to paediatric specialist care and a urine sample should be sent for urgent microscopy and culture. These infants should be managed in accordance with the recommendations for this age group in ‘Feverish illness in children’ (NICE clinical guideline 47).		
**Urine testing strategy for infants and children 3 months or older but younger than 3 years**		
Urgent microscopy and culture is the preferred method for diagnosing UTI in this age group; this should be used where possible.		
If the infant or child has specific urinary symptoms	Urgent microscopy and culture should be arranged and antibiotic treatment should be started. When urgent microscopy is not available, a urine sample should be sent for microscopy and culture, and antibiotic treatment should be started	
If the symptoms are non-specific to UTI	• For an infant or child with a high risk of serious illness: the infant or child should be urgently referred to a paediatric specialist where a urine sample should be sent for urgent microscopy and culture. Such infants and children should be managed in line with ‘Feverish illness in children’ (NICE clinical guideline 47)	
	• For an infant or child with an intermediate risk of serious illness: if the situation demands, the infant or child may be referred urgently to a paediatric specialist. For infants and children who do not require paediatric specialist referral, urgent microscopy and culture should be arranged. Antibiotic treatment should be started if microscopy is positive. When urgent microscopy is not available, dipstick testing may act as a substitute. The presence of nitrites suggests the possibility of infection and antibiotic treatment should be started. In all cases, a urine sample should be sent for microscopy and culture	
	• For an infant or child with a low risk of serious illness: microscopy and culture should be arranged. Antibiotic treatment should only be started if microscopy or culture is positive	
**Urine-testing strategies for children 3 years or older**		
Dipstick testing for leukocyte esterase and nitrite is diagnostically as useful as microscopy and culture, and can safely be used.		
If both leukocyte esterase and nitrite are positive	The child should be regarded as having UTI and antibiotic treatment should be started. If a child has a high or intermediate risk of serious illness and/or a history of previous UTI, a urine sample should be sent for culture	
If leukocyte esterase is negative and nitrite is positive	Antibiotic treatment should be started if the urine test was carried out on a fresh sample of urine. A urine sample should be sent for culture. Subsequent management will depend upon the result of urine culture	
If leukocyte esterase is positive and nitrite is negative	A urine sample should be sent for microscopy and culture. Antibiotic treatment for UTI should *not* be started unless there is good clinical evidence of UTI (for example, obvious urinary symptoms). Leukocyte esterase may be indicative of an infection outside the urinary tract, which may need to be managed differently	
If both leukocyte esterase and nitrite are negative	The child should not be regarded as having UTI. Antibiotic treatment for UTI should not be started, and a urine sample should not be sent for culture. Other causes of illness should be explored	
**Guidance on the interpretation of microscopy results**		
**Microscopy results**	**Pyuria positive**	**Pyuria negative**
**Bacteriuria positive**	The infant or child should be regarded as having UTI	The infant or child should be regarded as having UTI
**Bacteriuria negative**	Antibiotic treatment should be started if clinically UTI	The infant or child should be regarded as not having UTI
**Indication for culture; urine samples should be sent for culture:**		
• in infants and children who have a diagnosis of acute pyelonephritis/upper urinary tract infection		
• in infants and children with a high to intermediate risk of serious illness		
• in infants and children younger than 3 years		
• in infants and children with a single positive result for leukocyte esterase or nitrite		
• in infants and children with recurrent UTI		
• in infants and children with an infection that does not respond to treatment within 24–48 h, if no sample has already been sent		
• when clinical symptoms and dipstick tests do not correlate		

The NICE guidance does not specifically give recommendations to laboratories on how they should process and report children's urine culture results to clinicians. Public Health England (PHE) standards for microbiology investigation state ‘Generally, a pure growth of between 10^7^–10^8^ c.f.u. l^− 1^ is indicative of UTI’, but ‘Colony counts of ≥ 10^6^ c.f.u. l^− 1^ of a single species may be diagnostic of UTI’. The PHE standards tables on how to report urine specimens go into more detail and indicate that a growth of 10^6^ c.f.u. l^− 1^ (>1000 c.f.u. ml^− 1^) with a single organism should be considered a possible UTI, and >10^7^ c.f.u. l^− 1^ (10 000 c.f.u. ml^− 1^) with a predominant organism in a mixed growth of two organisms should be considered a probable UTI, in the presence of symptoms and white blood cells. One of the other recommendations of NICE that has implications for laboratory reporting is ‘Infants and children with atypical UTI [this includes infection with non-*Escherichia coli* bacteria (Jantunen *et al.*, 2001)] should have ultrasound of the urinary tract during the acute infection’ (Box 2) (NICE, 2007).

**Table:** Box 2 Extract from NICE 2007 guidance recommendations for imaging infants and children, and definitions of recurrent and atypical UTI (NICE guidance Table 6.12)

**NICE guidance for imaging infants and children**	
Infants and children with atypical UTI should have ultrasound of the urinary tract during the acute infection to identify structural abnormalities of the urinary tract such as obstruction. This is to ensure prompt management	• For infants aged younger than 6 months with first-time UTI that responds to treatment, ultrasound should be carried out immediately if atypical or recurrent, or otherwise within 6 weeks of the UTI
	• For infants and children 6 months or older with first-time UTI that responds to treatment, routine ultrasound is not recommended unless the infant or child has atypical UTI
	• Infants and children who have had a lower UTI should undergo ultrasound (within 6 weeks) only if they are younger than 6 months or have had recurrent infections
	• A DMSA scan 4–6 months following the acute infection should be used to detect renal parenchymal defects
	• If the infant or child has a subsequent UTI while awaiting DMSA, the timing of the DMSA should be reviewed and consideration given to doing it sooner
	• Routine imaging to identify VUR is not recommended for infants and children who have had a UTI, except in specific circumstances
NICE Definitions of atypical	• Infection with non-*E. coli* organisms
UTI	• Seriously ill (for more information refer to ‘Feverish illness in children’)
	• Poor urine flow
	• Abdominal or bladder mass
	• Raised creatinine
	• Septicaemia
	• Failure to respond to treatment with suitable antibiotics within 48 h
NICE Definitions of recurrent UTI	• Two or more episodes of UTI with acute pyelonephritis/upper UTI, or
	• one episode of UTI with acute pyelonephritis/upper UTI plus one or more episode of UTI with cystitis/lower UTI, or
	• three or more episodes of UTI with cystitis/lower UTI

We aimed to 1) audit the extent of the availability of urgent urine microscopy for children in hospital and in primary care, 2) audit what methods are used to culture children's urine, 3) audit how children's urine culture results are routinely reported and 4) determine, in a subset of laboratories, whether NICE guidance had led to a change in numbers of urine specimens from children under three years being submitted and/or reported. This audit scope allowed us to determine whether laboratories facilitate compliance with NICE recommendations. The audit formed part of a Healthcare Quality Improvement Partnership (HQIP) multi-centre audit (HQIP NCA 075) (HQIP, 2013) that focused on three key clinical themes of the childhood UTI NICE guidance; improving the rate of diagnosis, careful clinical evaluation to identify ‘high risk’ children and selective use of follow-up investigations.

## Methods

### Questionnaire development

The audit questionnaire (Appendix 1) was developed by clinical microbiologists and a consultant paediatrician and piloted several times with three laboratories for ease of completion and feasibility of data entry. The 14 questions were phrased to enable us to determine whether the microscopy, culture and antibiotic susceptibility service laboratories provided met the recommendations within the NICE UTI guidance (Boxes 1 and 2) and the UK Standards for Microbiology Investigations (NICE, 2007; The Standards Unit, Public Health England UK, 2014). This included how laboratories defined and reported childhood UTI and how laboratories reported non-*E. coli* bacteria.

To determine urine submission rates, laboratories in south-west England were asked to provide the number of all urine specimens submitted to them between 2003 and 2011 from children under 3 years of age (excluding Special Care Baby units and neonatal intensive care units) and the number of positive urines in this age group in whom antibiotic susceptibility results were reported. Laboratories were asked to report any changes in laboratory methodology or reporting protocols.

### Questionnaire distribution

The audit questionnaire was emailed with an explanatory letter to the managers of all 164 microbiology laboratories in England with Clinical Pathology Accreditation (CPA) in May 2011. The letter and questionnaire both stated that the aim of the HQIP audit was to determine whether laboratories identify and report organisms, in line with the 2007 NICE guidance, allowing children with non-*E. coli* UTI to be identified and then investigated further by clinicians. We asked that a biomedical scientist or consultant medical microbiologist who was responsible for childhood urine culture and susceptibility reports complete the questionnaire. An email reminder was sent in June 2011. As the initial response rate was under 50 %, a personalised letter was sent to all microbiology consultants in August 2011. In October the remaining 35 non-responding laboratories were phoned to confirm that they processed childhood urine specimens and therefore should be included in the non-responders.

### Data analysis

The number of responses and proportion of different responses to each question were tabulated. Data were cleaned by one of the research team, and where laboratories had ticked more than one box for an answer, comments given by participants were used to determine which answer was recorded. The 95 % confidence intervals (CI) were obtained by using the binomial distribution, with the number of responses as parameters thereto.

We assumed that the number of children under three in each area where urine submission data were collected was stable over time. For each laboratory, negative binomial or Poisson regression models, as appropriate, were fitted to the number of urines and number of positive specimens in which an antibiotic susceptibility was reported. For each laboratory except laboratory 4, the covariates in the model were year, a dichotomous variable indicating 2008 and later, and a variable indicating the number of years after 2007, which allowed for trend pre-guidance, a change between pre- and post-guidance in the number (level) of the outcome between what it would have been in 2008 without guidance and its prediction in 2008 as a result of the guidance, and change in the trend from 2008 onwards, respectively. Since there were only three observations for laboratory 4 in the post-guidance period, the only covariate in the model was a variable equal to the year less 2008, so that year numbering began from 1. Statistical significance was determined by means of the likelihood ratio test. For laboratory 4, the significance of the trend was determined, whereas for all the other laboratories the *P* values for significance of change in trend and change in level were obtained. All analyses were performed in stata version 13.0 (StataCorp, 2011).

### Ethics

As this was an audit it did not require ethical approval but it was undertaken in line with information governance and Caldicott principles.

## Results

The questionnaire was sent to 164 laboratories; 14 of these did not process urines, five declined to take part due to lack of time, and we contacted 30 of the 35 non-responding laboratories, all of which confirmed that they did routinely perform urine cultures from children. Thus, 73 % (110/150) of laboratories undertaking childhood urine culture returned completed questionnaires. Non-responding laboratories were not atypical; they were from across England serving both rural and urban communities and included both private and NHS laboratories. Seventy-five per cent of laboratories (83/110) returned data on the number of urines submitted from children under 16 years in 2010 and population served. There was a wide range of urine submissions from children to laboratories from 438 to 36 400; this equated to a range of 1 to 161/1000 total population. Six laboratories in the South West returned detailed data on laboratory submissions.

### Compliance with NICE recommendations for urgent microscopy

The majority of the laboratories (88 %, 97/110) undertook urgent microscopy for hospitalized children; 18 % only during office hours, 25 % during office and extended hours and 57 % during all hours. Only 54 % of laboratories (59) also processed urgent specimens from children in general practice.

### Compliance with national recommendations for cut off for a positive culture, determined by volume of urine cultured and reporting policy

PHE operating standards state that 10^6^ c.f.u. l^− 1^ (>1000 c.f.u. ml^− 1^) with a single species may be diagnostic of UTI, or >10^7^ c.f.u. l^− 1^ (10 000 c.f.u. ml^− 1^ *)* predominant organism in a mixed growth of two isolates should be considered a possible UTI, in the presence of symptoms and significant numbers of white blood cells (The Standards Unit, Public Health England, 2014). Sixty-one per cent of laboratories (CI 52–70 %, 67/110) reported that they cultured 1 μl of urine from children, 10 % 2–3 μl, 5 % 5 μl, 10 % 10 μl, 5 % 20–100 μl, 7 % used a filter paper method and 2 % used an automated method. Fifty-nine per cent of laboratories (CI 49–68 %, 65/110) reported 10^7^ c.f.u. l^− 1^ (10 000 c.f.u. ml^− 1^) as significant, 19 % (21/110) reported 10^6^ c.f.u. l^− 1^ (>1000 c.f.u. ml^− 1^) as significant and 15 % (16/110) reported 10^8^ c.f.u. l^− 1^ (10^5^ c.f.u. ml^− 1^) as significant. Eighteen per cent of laboratories (20/110) specifically commented that they would only report the lower colony counts if in pure growth or accompanied by significant white cells.

Laboratories reported much lower counts from suprapubic aspirates; 42 % (CI 32–52 %, 46/110) reported 10^6^ c.f.u. l^− 1^ (1000 c.f.u. ml^− 1^), 35 % (39/110) reported any growth or 100 c.f.u. ml^− 1^ (10^5^ c.f.u. l^− 1^) and 20 % (22/110) 10^7^ c.f.u. l^− 1^ (10 000 c.f.u. ml^− 1^). Only one laboratory reported 10^8^ c.f.u. l^− 1^ (10^5^ c.f.u. ml^− 1^).

Seventy-one per cent of laboratories (CI 61–79 %, 78/110) reported a mixed growth of 10^8^ c.f.u. l^− 1^ (10^5^ c.f.u. ml^− 1^) if there was a single predominant organism over 10^7^ c.f.u. l^− 1^ (10 000 c.f.u. ml^− 1^); another 7 % (8/110) reported these if the urine was a repeat sample, if there were significant numbers of white blood cells or if clinically indicated.

### Are laboratories distinguishing and reporting coliforms as either *E. coli* or non-*E. coli*?

For the routine identification of uropathogens, 81 % of laboratories (89/110) used chromogenic agar, which allows the easy differentiation of *E. coli* from other non-*E. coli* coliforms (Aspevall *et al.*, 2002). Eighteen per cent of laboratories (20/110) used CLED (cysteine-, lactose- and electrolyte-deficient) agar and 0.9 % (1/110) used MacConkey agar; these do not allow differentiation of *E. coli* from other lactose-fermenting coliforms (Aspevall *et al.*, 2002). *E. coli* was reported by its species name by 83 % (91/110) of laboratories; 17 % laboratories (19/110) only reported it as a coliform. *Enterobacteriaceae* that were not *E. coli* (e.g. *Klebsiella*, *Serratia*, *Enterobacter*, *Proteus*) were reported only as coliforms (rather than non-*E. coli* coliforms) by 61 % (67/109) of laboratories (seven of these reported *Proteus* spp. when present); 37 % (40/109) reported to genus or species level dependent on the organism and 1.8 % (2/109) reported these as coliforms other than *E. coli*. Eighty-five per cent of laboratories (17/20) who used CLED routinely reported *Enterobacteriaceae* that were not *E. coli* as coliforms, compared with 50 % of laboratories (43/86) who used chromogenic agar for primary culture.

When reporting a multi-resistant non-*E. coli* coliform, most reported to species level (84 %, 92/110) or genus level (8.7 %); only 9 % (10/110) of laboratories reported these as coliforms. When a non-*E. coli* was not an *Enterobacteriaceae*, all laboratories reported these to genus or species level. Two (1.8 %) laboratories routinely added a standardized comment to the urine report if the organism isolated was a non-*E. coli* indicating that the child may need further investigation; one other laboratory (0.9 %) linked all reports to local UTI guidance.

### Laboratory audit of urine submissions and UTIs diagnosed in children under 3 years old in south-west England (Fig. 1, Table 1)

**Table 1.** Trends and trend differences for each laboratory between 2003 and 2011 in (a) number of urines submitted from children under 3 years and (b) number of positive specimens reported by laboratories with antibiotic susceptibility results [including those when growth was below 10^8^ c.f.u. l^− 1^ (10^5^ c.f.u. ml^− 1^)] from children under 3 years

**Lab**	**Trend pre-guidance (%) (95 % CI)**	**Change in trend from pre- to post-guidance (%) (95 % CI)**	**Trend post-guidance (%) (95 % CI)**	***P*** **value for change**	**Level change (%) (95 % CI)**	***P*** **value for level change**
**(a) Urines submitted**						
1	− 4.7 ( − 9.7, 0.6)	8.4 ( − 1.4, 19.1)	3.3 ( − 4.4, 11.7)	0.12	− 6.7 ( − 23.4, 13.7)	0.4
2	− 1.4 ( − 8.9, 6.7)	1.7 ( − 6.4, 10.5)	0.3 ( − 2.2, 2.9)	0.7	− 1.6 ( − 8.6, 5.8)	0.7
3	− 7.2 ( − 25.3, 15.2)	16.4 ( − 7.1, 46.0)	8.0 (1.1, 15.3)	0.20	3.6 ( − 15.1, 26.2)	0.7
5	1.3 ( − 1.5, 4.2)	− 6.3 ( − 10.9, − 1.4)	− 5.1 ( − 8.9, − 1.0)	0.03	− 9.2 ( − 18.1, 0.6)	0.5
6	− 2.4 ( − 5.6, 1.0)	− 1.0 ( − 6.5, 4.9)	− 3.3 ( − 7.8, 1.3)	0.7	27.0 (12.5, 43.4)	0.005
**(b) Number of positives reported with susceptibility result**						
1	− 6.1 ( − 14.0, 2.6)	38.3 (20.8, 58.3)	29.8 (17.2, 43.8)	0.001	24.7 ( − 8.4, 69.9)	0.8
2	25.0 (3.4, 51.1)	− 14.7 ( − 30.0, 4.1)	6.7 (0.7, 13.0)	0.12	− 11.5 ( − 25.2, 4.7)	0.07
3	18.8 ( − 4.1, 47.2)	− 18.1 ( − 34.5, 2.3)	− 2.7 ( − 8.3, 3.2)	0.09	24.1 (3.5, 48.8)	0.04
5	0.6 ( − 4.9, 6.3)	2.2 ( − 11.4, 8.0)	− 1.6 ( − 9.4, 6.8)	0.7	− 8.1 ( − 25.1, 12.8)	0.6
6	8.7 (12.1, 35.5)	− 8.6 ( − 15.0, − 1.7)	− 0.7 ( − 5.8, 4.8)	0.04	48.5 (27.6, 72.3)	0.004

A negative percentage trend indicates submissions were decreasing, and a positive percentage indicates submissions were increasing; significant changes in bold type.

Laboratory 4 is not represented as we only had post-guidance data.

Six laboratories in the South West returned urine submission data; laboratories 2, 3 and 4 were only able to collect data from 2006, 2007 and 2009, respectively.

Only laboratory 6 showed a significant increase in submission of urine specimens from children under 3 years and a significant increase in urines reported with antibiotic susceptibilities between the pre-2007 level in the absence of guidance and the 2008 level following guidance (27.0 % increase in submission, 95 % CI 12.5–43.4 %, *P* value = 0.005; 48.5 % increase in positive reports, 95 % CI 27.6–72.3 %, *P* = 0.004). Laboratory 1 had no increase in submissions, but had a significant increase in positives reported; this increase occurred after 2010 when the laboratory indicated that they had a change in reporting policy and started reporting antibiotic susceptibility for organisms within a mixed growth if there was a predominant organism greater than 10^7^ c.f.u. l^− 1^ (10^4^ c.f.u. ml^− 1^). Laboratory 3 had no significant increase in submission or an increasing trend, but the level of positives increased significantly after the guidance was published (24.1 % increase in level, 95 % CI 3.5–48.8 %, *P* = 0.04); however, there was no change in testing or reporting policy. Laboratories 2 and 5 had no significant increases; furthermore, laboratory 5 had a significant decreasing trend in submissions (percentage change in trend slope lab 5 − 6.3 %, 95 % CI − 10.9 to − 1.4 %, *P* = 0.03).

Laboratory 4, with 3 years of data from 2009 to 2011, demonstrated a significant increase in specimen submission (8.3 %, < 0.001); however, the number of positives reported with antibiotic susceptibility results decreased non-significantly by 6.7 % (*P* = 0.10); there was no change in testing or reporting policy.

## Discussion

### Main findings

Forty-six per cent of laboratories are not undertaking urgent microscopy in general practice paediatric urines and 61 % are unable to reliably diagnose a UTI with a colony count of under 10^8^ c.f.u. l^− 1^ (10^5^ c.f.u. ml^− 1^); 61 % (67/109) are not fully identifying most non-*E. coli* coliforms and, therefore, are not giving enough information on reports for clinicians to be able to refer children in whom an *Enterobacteriaceae* other than *E. coli* has been isolated. Only 22 % (24/110) of laboratories comply with both the recommendations for urgent microscopy for all children and clearly differentiating non-*E. coli* coliforms; only 2.7 % (3/110) of laboratories also cultured a 5 μl volume of urine allowing them to diagnose an infection with 10^6^ c.f.u. l^− 1^ (>1000 c.f.u. ml^− 1^) with any confidence. Our audit of laboratory submissions showed that for five of the six laboratories the guidance has not significantly increased urine submissions or diagnosis of UTIs in children less than three years.

### Strengths and limitations

The questionnaire survey had a very good return and, therefore, probably reflects current laboratory practice across English laboratories. The participants reported standard operating procedures; however, this cannot replace a prospective audit of clinical cases and their outcomes, including whether children with atypical UTIs caused by non-*E. coli* received appropriate imaging tests. Furthermore, we made no attempt to identify the quality of the sample received and the quality of the request information provided which would have also required a more in-depth audit; these two factors may have a significant effect on the reliability and interpretation of results. Such in-depth audits would be very difficult to undertake on a large number of laboratories. The urine submission audit was only undertaken in the South West and due to computer changes, only three laboratories were able to provide submission data for more than one year pre-guidance, making interpretation more difficult. This reflects ongoing problems of using routinely generated data to monitor the effect of guidance on laboratory practice and the difficulties of determining whether NICE guidance has increased the numbers of childhood UTIs diagnosed.

### Where this fits in

NICE guidance indicates that urgent microscopy is the preferred diagnostic method in children under 3 years, as the diagnostic performance of urine dipstick nitrite and leukocyte esterase tests, in particular the likelihood ratios, is poor (NICE 2007). In all children under 3 months and those under 3 years with risk of serious illness, referral to the hospital setting is advised. However, NICE indicates that some infants or children with intermediate risk of serious disease may be managed in primary care where urgent microscopy is preferable. Although they suggest that urine dipstick can be used in children with less serious illness, elsewhere in the guidance they state ‘that a combination of a positive leucocyte esterase with positive nitrite has the highest LR+ and is the most useful dipstick test for ruling in UTI. However, a negative result for either leucocyte esterase or nitrite has the highest LR −  and will be most useful in excluding UTI. It is important to note that in children younger than 2 years the dipsticks are less reliable in both scenarios’. (NICE, 2013). Thus, ideally we recommend that one would have a system where there is widespread access to urgent microscopy by Primary Care, given the unreliability of dipsticks in the younger children.

Improved access to urgent microscopy by Primary Care may be a challenge with the ongoing reorganization of laboratory services moving to larger, more centralised laboratories well away from the point of care. Any service contracts with microbiology laboratories need to ensure that microscopy provision is available in some way at the point of care. The current relative lack of access to urgent microscopy services may delay appropriate treatment and, due to the unreliability of urine dipticks in children under 3 years, may result in general practitioners having to refer these children in whom they suspect a UTI to hospital; this may be neither cost effective nor appropriate for the child and family. Alternatively, the general practitioner may treat the patient based on symptoms and/or dipstick alone, which is less than ideal.

The NICE guidance defines UTI by a combination of clinical features and the presence of bacteria in the urine. It indicates that typically a UTI is caused by a single organism, which is present in a high concentration, usually greater than 10^8^ c.f.u. l^− 1^ (10^5^ c.f.u. ml^− 1^) (Kass, 1962), but that it is possible to have an infection which gives rise to a lower colony count or to a mixed growth (NICE, 2007). PHE standards for microbiology investigations and European guidance (Kouri *et al.*, 2000) indicate that a pure growth of over 10^8^ c.f.u. l^− 1^ (10^5^ c.f.u. ml^− 1^) is generally indicative of UTI; a pure or predominant growth of an organism at over 10^7^ c.f.u. l^− 1^ (10^4^ c.f.u. ml^− 1^) may be considered significant if there is pyuria and the child is symptomatic (The Standards Unit, Public Health England, 2014). Both sets of guidance indicate that colony counts of 10^6^ c.f.u. l^− 1^ (>1000 c.f.u. ml^− 1^) of a single species may be diagnostic of UTI in voided urine (mid-stream or clean-catch urine rather than a nappy urine) in the presence of symptoms. The PHE standard methods state that if a 1 μl or 2 μl inoculum or filter paper method is used, the method is only sensitive for screening down to 10^7^ c.f.u. l^− 1^ (10^4^ c.f.u. ml^− 1^) (The Standards Unit, Public Health England, 2014); only 20 % of the laboratories surveyed who culture 5 μl or more would be able to diagnose an infection giving rise to 10^6^ c.f.u. l^− 1^ (>1000 c.f.u. ml^− 1^) with any confidence. The recent Diagnosis of Urinary Tract Infection in Young Children (DUTY) study (Downing *et al.*, 2012) cultured urine from unwell children under 5 years old with suspected infection. Only 2.2 % of 5000 children whose urine was cultured in a research laboratory using 5 μl aliquots and a spiral plating technique had confirmed UTI (Hay *et al.*, 2013); higher colony counts were more predictive of infection, but children with lower counts of 10^6^ c.f.u. l^− 1^ (>1000 c.f.u. ml^− 1^) and 10^7^ c.f.u. l^− 1^ (10^4^ c.f.u. ml^− 1^) were also considered to have UTI, although there were more in this group with another confirmed cause for their illness. If children have urine with lower counts the laboratory should probably request a repeat specimen, as the probability of UTI is increased by the isolation of the same organism from two specimens (Coulthard *et al.*, 2010). Lower counts are particularly relevant in urine specimens obtained by suprapubic aspiration (Kanellopoulos *et al.*, 2005; Hansson *et al.*, 1998) and all the laboratories except one reported lower colony counts as significant; 42 % reported 10^6^ c.f.u. l^− 1^ (>1000 c.f.u. ml^− 1^) as significant from these specimens. The PHE standards do not give specific recommendations for significance of counts from suprapubic aspirates but do state that ‘(SPA) is seen as the “gold standard” but is usually reserved for clarification of equivocal results from voided urine in infants and small children’ (The Standards Unit, Public Health England, 2014).

The 2013 NICE Urinary tract infection in infants, children and young people quality standard states that: ‘If a urinary tract infection is caused by a non-*E. coli* coliform or any other type of bacteria, there is an increased risk of serious underlying pathology. NICE guidance recommends that infants, children and young people (under 16 years) with atypical urinary tract infection (which includes infection with non-*E. coli* organisms) should have ultrasound of the urinary tract during the acute infection. It is therefore important that laboratory test reports differentiate between *E. coli* and non-*E. coli* organisms to identify whether further investigations are needed’ (NICE, 2007). Although 83 % of laboratories reported *E. coli* itself by name, we found the laboratories in our audit had an inconsistent approach to reporting of non-*E. coli* organisms in urines. Although non-*Enterobacteriaceae* were often reported to genus or species level, non-*E. coli* coliforms were frequently not identified and were reported as coliforms by 61 % of laboratories. This was a key finding of our study. Laboratories who did not use chromogenic agar were more likely not to report non-*E. coli* coliforms just as coliforms (75 %), although 50 % of laboratories who used chromogenic agar similarly reported these as coliforms. As children with non-*E. coli* coliforms are more likely to have unrecognized renal abnormalities these may go unrecognized if clinicians do not realize they should perform the renal imaging recommended for these patients by the NICE 2007 guidance (NICE, 2007; Jantunen *et al.*, 2001). In one international study of children with febrile UTI, renal scarring was found by scanning in 86 % of 56 children when the infecting organism was non-*E. coli* (vs 57 % of 213 children with *E. coli*, *P* < 0.0001), 73 % of children with recurrent UTI and 77 % with severe reflux (Orellana *et al.*, 2004). Similarly, in a prospective study of 180 infants with acute pyelonephritis by Jantunen *et al.* (2001), infants with non-*E. coli* infections (*Enterococcus faecalis*, *Enterobacterium cloacae*, *Klebsiella pneumoniae* and *Klebsiella oxytoca*) had a 3.4-fold relative risk of significant urinary tract abnormalities and thus obstructive uropathy (Jantunen *et al.*, 2001). Despite the NICE guidance, the 2014 national Standard Microbiology Investigations do not oblige laboratories to identify non-*E. coli* coliforms beyond coliform level (The Standards Unit, Public Health England, 2014). In line with childhood UTI NICE guidance and the quality standards (NICE, 2013), it would also be best practice for laboratories to indicate with a comment in the report to clinicians the need to consider renal imaging in children with non-*E. coli* UTI. In our audit only two laboratories added any such routine comment to reports of non-*E. coli* isolates.

**Fig. 1.**
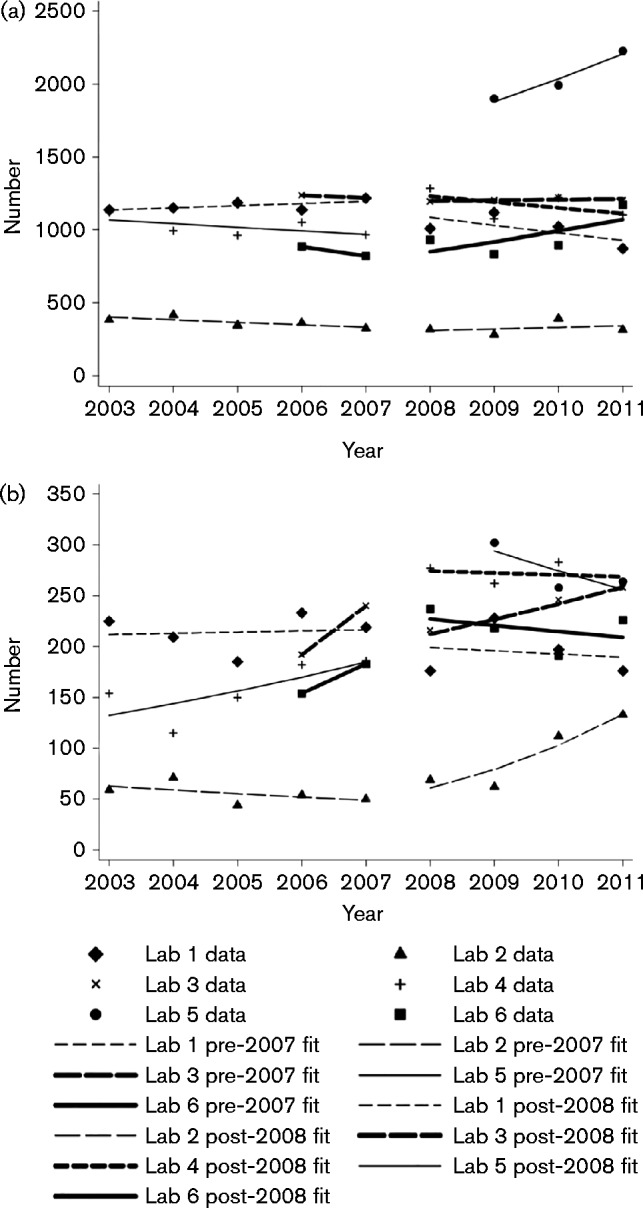
In each laboratory per calendar year in children under 3 years, (a) number of urine specimens submitted and (b) number of positive urines reported by laboratory with antibiotic susceptibility test results.

Previous work has shown a wide variation in urine specimen submission to laboratories (McNulty *et al.*, 2004) and this audit corroborates this, with a 100-fold variation in submission rate across the 83 laboratories returning data. Trends in the rates of urine submissions can be used as a measure of efforts by clinicians to improve the rate of diagnosis of UTI (McNulty *et al.*, 2008). Only one of our laboratories with data pre- and post-2007 had any significant change in urine submission, suggesting NICE guidance has had little effect on submission of urines in children under three years. It is not possible to say whether the increase in submission of urines to laboratory 4 is due to the NICE guidance as this was published in 2007 and we do not have prior data from this laboratory. In two laboratories, the rise in the number of positives reported did not follow the number of urines submitted (Fig. 1). One possible explanation is the reported change in reporting protocol by one of these laboratories that they started reporting antibiotic susceptibility for organisms within a mixed growth if there was a predominant organism greater than 10^7^ c.f.u. l^− 1^ (10^4^ c.f.u. ml^− 1^). In the other case it is possible to speculate that urines for culture were submitted from children with a higher pre-test likelihood of UTI.

There are two major obstacles in the diagnosis of UTI in this age group: UTI symptoms in this age group overlap with symptoms of much more common viral infections and it is an onerous task to obtain urine samples from children with no bladder control. The DUTY study, which has enrolled 7000 children and has detailed urine results for over 5000, may be able to inform a more robust algorithm than currently in the NICE guidance to help clinicians identify a set of clinical criteria that if present increase the likelihood of UTI, thereby focusing efforts in obtaining urine samples to this group of children (Downing *et al.*, 2012; Hay *et al.*, 2013).

### Implications

Laboratory microscopy should be made available to support a reliable and rapid diagnosis of UTI for young children in both the primary and secondary care setting. Since NICE considers that children with non-*E. coli* coliform UTI need to be identified (NICE 2007) and this is now a NICE standard (NICE, 2013) the national microbiology standard method 41 (The Standards Unit, Public Health England, 2014) should be changed to include this recommendation. Laboratories should review their laboratory Standard Microbiology Investigations and reporting for UTI and ensure it is in line with both NICE guidelines and the national standards. Laboratories could encourage referral of children with non-*E. coli* UTI for imaging by adding a routine comment to reports of non-*E. coli* isolates. NICE guidance needs more rigorous implementation through all relevant professional societies.

## Appendix A1. HQIP Paediatric UTI Laboratory Audit 2011

We do hope that you can take the time to complete this audit funded by Healthcare Quality Improvement Partnership (HQIP). The NICE guidance on diagnosis and management of childhood UTI was published in 2007.

The purpose of this laboratory audit is to evaluate the implementation of a key laboratory aspect of the guidance. NICE identified infections with non-*E. coli* organisms as a risk factor for kidney abnormalities. The guidance recommends that all infants and children with non-*E. coli* UTI should have renal imaging.

This audit aims to determine if laboratories examine urines in line with NICE and identify and report organisms to allow children with non-***E. coli*** UTI to be identified and then investigated further by clinicians.

Dr Cliodna McNulty Dr Lyda Jadresic

Head, HPA Primary Care unit Consultant Paediatrician, Gloucestershire Royal Hospital

1. Do you process urine specimens from children in general practice? YES NO

2. Do you undertake urgent microscopy of urine specimens from children in general practice? YES NO

3. Do you process urine specimens from hospital paediatric patients? YES NO

4. Do you undertake urgent microscopy of urine specimens from hospital paediatric patients? YES NO

5. Comments on times urgent microscopy service available ..............................................................................

6. What volume of a clean catch urine do you culture from a child under 16 years old?

1 μl 5 μl 10 μl 100 μl Other: Please state................................................................................

7. What cut-off in c.f.u. do you report in children < 16 years as significant in a clean catch urine from a child under 16 years old?

10^3^ c.f.u. ml^− 1^ 10^4^ c.f.u. ml^− 1^ 10^5^ c.f.u. ml^− 1^ Other:....................................

8. What cut-off in c.f.u. do you report in children < 16 years as significant in a supra-pubic aspirate in a child under 16 years old

10^3^ c.f.u. ml^− 1^ 10^4^ c.f.u. ml^− 1^ 10^5^ c.f.u. ml^− 1^ Other:....................................

9. Do you report as significant a mixed growth 10^5^ c.f.u. ml^− 1^ if there is a single predominant organism >10^4^ c.f.u. ml^− 1^? YES NO Other:...................................................................

10. How do you routinely identify urine isolates? Chromogenic agar CLED Other:

Please state......................... .............. ....................... .........................................................................................

11. How would you report an *E. coli*? ***E. coli*** or coliform?

12. How would you routinely report an organism that is not *E. coli*? First what about a coliform that is not *E. coli*?

Coliform Non-***E. coli*** coliform to genus level to species level Other:

Please specify how you report?......................................................................................................................

13. How would you routinely report a multi-resistant coliform that is not *E. coli*?

Coliform Non-***E. coli*** coliform to genus level to species level Other:

Please specify how you report?.......................................................................................................................

14. How would you routinely report an organism that is not a coliform?

To genus level to species level Other:

Please specify how you report?.......................................................................................................................

15. *Do you **routinely** add any **standardized** comments to the report if the organism isolated is a non-E. coli?*

YES NO Other: Please state:.......................................................................................

Name of laboratory.............................................................................Date.......................................................

16. Name of individual completing questionnaire in case of queries.......................................................................

Position Contact telephone email........................................................
